# Cryopreservation of Ovarian and Testicular Tissue and the Influence on Epigenetic Pattern

**DOI:** 10.3390/ijms241311061

**Published:** 2023-07-04

**Authors:** Tom Trapphoff, Stefan Dieterle

**Affiliations:** 1 Dortmund Fertility Centre, 44135 Dortmund, Germany; dieterle@kinderwunschzentrum.org; 2 Division of Reproductive Endocrinology and Infertility, Department of Obstetrics and Gynecology, Witten/Herdecke University, 44135 Dortmund, Germany

**Keywords:** epigenetic, genomic imprinting, ovarian tissue cryopreservation, testicular tissue cryopreservation, medically assisted reproduction

## Abstract

Ovarian tissue cryopreservation (OTC) or testicular tissue cryopreservation (TTC) are effective and often the only options for fertility preservation in female or male patients due to oncological, medical, or social aspects. While TTC and resumption of spermatogenesis, either in vivo or in vitro, has still be considered an experimental approach in humans, OTC and autotransplantation has been applied increasingly to preserve fertility, with more than 200 live births worldwide. However, the cryopreservation of reproductive cells followed by the resumption of gametogenesis, either in vivo or in vitro, may interfere with sensitive and highly regulated cellular processes. In particular, the epigenetic profile, which includes not just reversible modifications of the DNA itself but also post-translational histone modifications, small non-coding RNAs, gene expression and availability, and storage of related proteins or transcripts, have to be considered in this context. Due to complex reprogramming and maintenance mechanisms of the epigenome in germ cells, growing embryos, and offspring, OTC and TTC are carried out at very critical moments early in the life cycle. Given this background, the safety of OTC and TTC, taking into account the epigenetic profile, has to be clarified. Cryopreservation of mature germ cells (including metaphase II oocytes and mature spermatozoa collected via ejaculation or more invasively after testicular biopsy) or embryos has been used successfully for many years in medically assisted reproduction (MAR). However, tissue freezing followed by in vitro or in vivo gametogenesis has become more attractive in the past, while few human studies have analysed the epigenetic effects, with most data deriving from animal studies. In this review, we highlight the potential influence of the cryopreservation of immature germ cells and subsequent in vivo or in vitro growth and differentiation on the epigenetic profile (including DNA methylation, post-translational histone modifications, and the abundance and availability of relevant transcripts and proteins) in humans and animals.

## 1. The Epigenome

**Figure 1:**
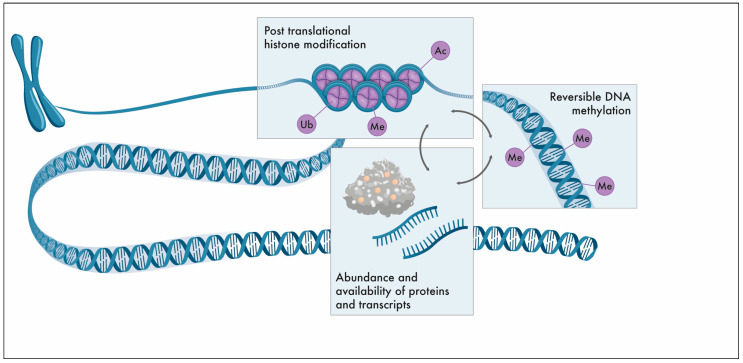
Scheme of epigenetic control. The epigenome includes reversible DNA methylation, post-translational histone modifications (PTMs), and the abundance and availability of relevant transcripts and proteins to establish or maintain DNA methylation. Methylation (Me), acetylation (Ac), and ubiquitylation (Ub).

**Figure 2:**
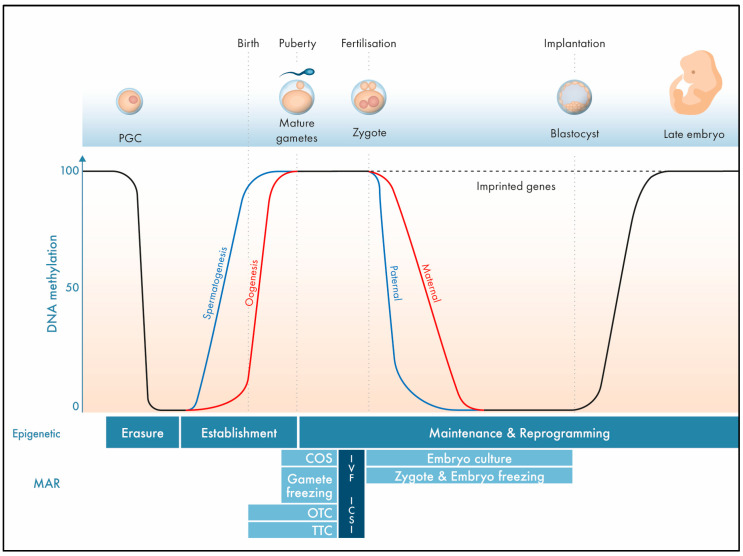
DNA methylation pattern in gametogenesis and early embryogenesis. The DNA methylation pattern is erased in primordial germ cells (PGC) followed by subsequent de novo establishment of the germ cell-specific DNA methylation profile for imprinted genes and gDNA. During early embryogenesis, global DNA demethylation occurs in the zygote and growing embryo. During embryogenesis, cell- and tissue-specific de novo gDNA reprogramming is established. Methylation pattern of imprinted genes is maintained after fertilisation and embryogenesis.

Exactly 70 years ago, DNA was described as the universal carrier of genetic information through the pioneering work of Francis Crick, James Watson, and Rosalind Franklin. However, gene expression relies on more than just the nucleobase sequence; epigenetic factors control cellular functions at a superimposed level without affecting the genetic code itself. The epigenome includes reversible DNA methylation, post-translational histone modifications (PTMs), and the abundance and availability of relevant transcripts and proteins to establish or maintain DNA methylation (Figure 1). It is not surprising that epigenetic control is crucial for cell and tissue differentiation, reaction to exogenous and endogenous influences, sex chromosome dosage compensation, or fertilisation and embryogenesis [1]. Epigenetic modifications are not rigid during the entire life cycle; rather, they are subjected to dynamic reprogramming. In brief, early in life, the entire somatic DNA methylation pattern is erased in primordial germ cells, with subsequent de novo establishment of the germ cell-specific DNA methylation profile. In males, global DNA methylation (gDNA) is completed in pachytene-stage spermatocytes; in females, gDNA is established in mature post-menarche oocytes [2,3]. To establish totipotency during early embryogenesis, gDNA demethylation occurs actively in paternally inherited DNA and passively in maternally inherited DNA in the zygote and growing embryo at each cell division (Figure 2). Later, during embryogenesis, cell and tissue-specific de novo gDNA reprogramming is established. 

Imprinted genes represent unique DNA sequences within the genome. Genomic imprinting occurs oppositely in differentially methylated regions (DMR) in male and female germ cells, leading to mono-allelic gene expression after syngamy. This evolutionarily conserved mechanism is essential for metabolic function and embryonic, placental, and postnatal development by expressing one parental allele and inactivating the other parental allele. De novo establishment of parental-specific DNA methylation takes place during gametogenesis in sperm and oocytes at specific CpG (cytosine–phosphate–guanine dinucleotide) sites; however, conversely to gDNA methylation, the methylation pattern of imprinted genes is maintained after fertilisation and embryogenesis. The progressive establishment of characteristic DNA methylation during gametogenesis depends on the de novo methyltransferases DNMT3A and/or DNMT3B/DNMT3L [4]. Several of the 200 known imprinted genes are organised into imprinted control regions (ICR). After oocyte-to-embryo transition, it is essential to distinguish between gDNA methylation, which becomes erased, and imprinted genes, where the methylation pattern is stable (Figure 2). To this end, a complex machinery is required to protect imprinted genes from demethylation in the early stages of life, which includes Maintenance DNA methyltransferase 1 (DNMT1), zinc finger protein 57 (ZFP57), MATER protein homologue (MATER), or STELLA. Many of these factors belong to the group of maternal effect genes (MEG) and are often stored in distinct compartments, such as the subcortical maternal complex (SCMC). The accumulation of these factors during gametogenesis and availability after fertilisation is essential for epigenetic control [2]. It is also noteworthy that post-translational histone modifications, mainly by acetylation, methylation, ubiquitylation of specific histone residues, or specific small non-coding RNAs, are also involved in this highly coordinated orchestra of epigenetic regulation [5,6]. Post-translational histone modifications also occur in a stage-specific manner and are essential for gene activation/silencing not just due to their ability to act as mediators between the enzymatic machinery and DNA itself. For instance, H3K4me3 is commonly associated with gene expression and cell differentiation, while H3K9me3 refers to heterochromatic DNA [7,8,9]. Thus, epigenetics is much more complex than simple DNA methylation itself, involving a dense network at different cellular levels. Disturbances due to cryopreservation or in vitro treatment may, therefore, interfere with the correct establishment and maintenance of the epigenome. To estimate any potential adverse effects of OTC or TTC, we must consider not just the methylation landscape but also factors located up- and downstream of DNA methylation.

## 2. Imprinting Disorders

A dozen clinical syndromes are known to be caused by imprinting disorders (e.g., Beckwith–Wiedemann syndrome (BWS), Silver–Russell syndrome (SRS), Prader–Willi syndrome (PWS), transient neonatal diabetes mellitus, recurrent miscarriages, and hydatidiform moles). For instance, total or partial loss of maternal methylation at the *SNURF-SNRPN* imprinting centre can lead to Angelman syndrome, maternal *ICR1* hypermethylation to Beckwith–Wiedemann syndrome, and paternal loss of *ICR1* methylation to Silver–Russell syndrome [10,11,12]. Aberrant methylation patterns can derive from genomic errors (e.g., DNA mutation/deletion/duplication) or epimutations themselves (e.g., gain/loss of DNA methylation patterns). As mentioned above, DNA methylation is embedded in a complex cellular network to establish stage-specific DNA methylation or to protect imprinted genes from demethylation. Therefore, genomic-based errors often lead to multi-locus imprinting disturbances (MLID), whereby one factor controls and regulates a broad spectrum of differently methylated regions. A genomic mutation in the *ZFP57* gene results in abnormal DNA methylation, as seen in patients with neonatal diabetes mellitus; mutations in the *NLRP* gene family (which includes *MATER* and *NLRP7*) result not only in miscarriages and hydatidiform moles but also imprinting disorders, such as Beckwith–Wiedemann syndrome or Silver–Russell syndrome, including the loss of DNA methylation in several DMRs [13,14,15,16]. 

Epigenetic DNA methylation is not only influenced indirectly by genomic errors, such as altered MEGs or components of the SCMC, but also directly by environmental stressors. Environmental exposure, such as in vitro culture, ovarian stimulation, or cryopreservation in early development might induce epimutations, as reported in different species [16,17,18,19,20]. Epimutations were not only reported in vitrified oocytes or embryos but also in later foetuses and placentas derived from cryopreserved embryos [20,21,22]. In humans, several studies also reported an increased risk of rare genomic imprinting disorders in children conceived via MAR techniques, including BWS, AS, PWS, and SRS [19,23,24]. It is also noteworthy that the use of MAR techniques is increasing globally during the last decades since the first successful in vitro fertilization (IVF) treatment in humans was performed in 1978 by Robert Edwards and Patrick Steptoe [25]. Overall, MAR consists of a broad spectrum of techniques including controlled ovarian stimulation (COS) and oocyte pick-up, in vitro fertilization or intracytoplasmic sperm injection (ICSI), in vitro embryo culture, and cryopreservation of gametes, zygotes, or embryos to support patients with the conception of a child in case of infertility, genetic predispositions, or fertility preservation, respectively (Figure 2, lower part) [26,27,28]. Nevertheless, it is still unclear whether MAR techniques per se or the impaired fertility background of the parents contributed to these rare genomic disorders in MAR children; however, epigenome alterations due to MAR treatment could be a possible mechanism. 

## 3. Ovarian Tissue Cryopreservation (OTC)

Cryopreservation of mature metaphase II (MII) oocytes, zygotes, or embryos is the common method to safeguard fertility in female patients after controlled ovarian stimulation and oocyte pick-up in MAR. Fertility preservation is primarily indicated in patients undergoing gonadotoxic radiotherapy or chemotherapy treatment, while other medical or social reasons (e.g., individual life planning) may also be of relevance. However, for women undergoing oncological treatment that cannot be delayed or prepubertal girls with no possibility of obtaining mature germ cells, OTC is the only option to preserve fertility. OTC can also be offered to female patients with benign ovarian diseases requiring ovariectomy and conditions with an increased risk of premature ovarian failure [29,30]. 

Ovarian tissue cryopreservation and transplantation were first performed in animal model systems several decades ago [31], while the first human live birth was reported in 2004 by Donnez and colleagues after controlled tissue freezing [32]. To date, more than 200 babies have been born worldwide after OTC. Before gonadotoxic cancer treatment or premature loss of the ovarian reserve, the ovarian cortex with dormant immature follicles and germinal vesicle (GV) oocytes of one or both ovaries are separated into small pieces of approximately 5 × 5 mm with a thickness of 1–2 mm prior to cryopreservation [29]. OTC can be performed either by slow freezing protocols or an ultra-fast vitrification technique. Slow freezing protocols are commonly used for OTC and nearly all live births reported used this technique. Nevertheless, in recent years, vitrification has also become a very promising approach for OTC, with just a few live births reported after vitrification and warming of ovarian tissue [33,34]. Both techniques require cryoprotective agents (CPA) to avoid the uncontrolled and detrimental formation of intracellular ice crystals during the cryopreservation process. Dimethyl sulfoxide (DMSO), glycerol, propanediol (PrOH), or ethylene glycol (EG) are used as permeating agents, and nonpermeating agents, such proteins, sugars, and other macromolecules, are used to achieve controlled cell dehydration and cellular CPA uptake. In both techniques, an optimised balance between CPA concentration and exposure time is necessary to minimise potential cytotoxic CPA stress. Slow freezing protocols typically use low CPA concentrations combined with a long exposure time during controlled freezing, while CPA exposure during vitrification is much shorter. Colligative combinations of two or more CPAs are used for vitrification; however, significantly higher CPA concentrations are necessary to prevent harmful ice crystal formation during the ultra-fast transition into a glass-like amorphous solid [35]. CPAs are known to have toxic effects on cellular structures and functions, and exposure to DMSO can lead to epimutations in different cell types, including DNA hypo- or hypermethylation, alterations in post-translational histone modifications, or misregulated expression of epigenetically relevant DNA methyltransferases [36,37,38]. 

Following cryopreservation, CPAs are removed after thawing/warming for slow freezing as well as for vitrification. It should be noted that, due to the higher CPA concentrations needed for vitrification, residual CPAs are higher in ovarian tissue after vitrification/warming compared with slow freezing and thawing [39]. Thus, ovarian tissue vitrification might pose a higher risk of cytotoxic CPA exposure compared with slow freezing protocols. 

**Figure 3:**
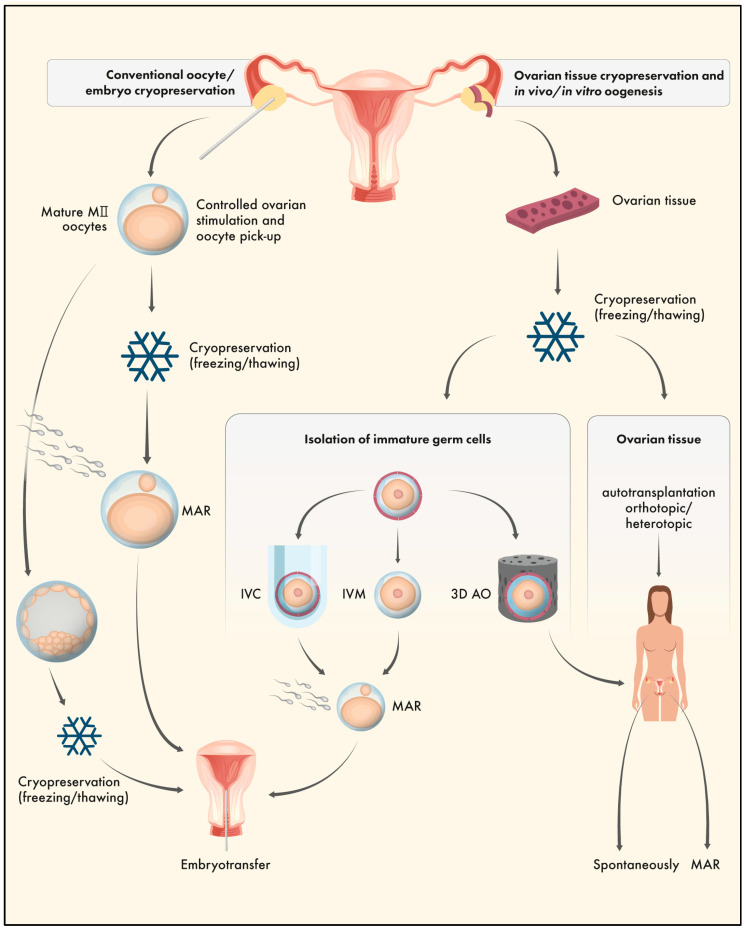
Strategies for fertility preservation in female patients. Conventional oocyte/zygote/embryo cryopreservation (left pathway) and ovarian tissue cryopreservation and in vivo/in vitro oogenesis (right pathway). Left pathway: Cryopreservation of mature oocytes or embryos after controlled ovarian stimulation and medically assisted reproduction techniques. Right pathway: Ovarian tissue cryopreservation followed by in vivo or in vitro folliculogenesis and oogenesis. After orthotopic or heterotopic transplantation, fertilisation can occur spontaneously or via medically assisted reproduction. Techniques to avoid the risk of remission of malignant cells including in vitro follicle culture (IVC), in vitro maturation of immature oocytes (IVM), and artificial ovaries (3D AO) followed by medically assisted reproduction or autotransplantation.

Several in vivo or in vitro options are available to resume folliculogenesis and oogenesis after OTC, with ovarian tissue autotransplantation still being the method of choice (Figure 3). After orthotopic or heterotopic transplantation, endogenous functionality is commonly restored after up to six months, and resumption of folliculogenesis and oogenesis can occur in vivo. Mature MII oocytes are available for fertilisation either spontaneously (orthotopic autotransplantation) or via MAR techniques (heterotopic autotransplantation). Besides in vivo strategies, in some cases, in vitro treatment is necessary to avoid the risk of remission of malignant cells or to enhance the activation of resting follicles after ovarian tissue autotransplantation. In vitro culture of immature follicles (IVC), in vitro maturation of immature oocytes (IVM), artificial ovaries including isolated follicles enclosed in extracellular matrix tissue and in vivo growth (AO), or in vitro activation of immature follicles (IVA) prior to autotransplantation afford new strategies to preserve female fertility [30]. Most human in vitro techniques are still experimental; however, live births have been reported after IVA in patients with idiopathic primary ovarian insufficiency [33]. In vitro follicle culture systems are options when tissue autotransplantation is contraindicated. Healthy live births were reported in animal models after IVC of immature follicles several years ago [40]; however, human IVC is still challenging due to different culture conditions including growth factors and hormones, or simply due to unequal follicle sizes between species [41]. Nevertheless, human IVC is a promising option and progress was reported by McLaughlin and colleagues [42]. Here, fertilisable human MII oocytes were obtained after complete in vitro growth and maturation. The translation of these techniques from animal models to humans is ambitious and further research is needed; however, these studies are also indispensable to understanding the direct and long-term effects of the cryopreservation of immature germ cells combined with interrupted gametogenesis.

Overall, OTC and subsequent folliculogenesis and oogenesis can preserve fertility in women facing gonadotoxic treatment, premature loss of the ovarian reserve due to benign conditions, or in prepubertal girls, regardless of the cryopreservation protocols applied. Autotransplantation of ovarian tissue is commonly performed; however, in vitro techniques are promising, particularly when tissue autotransplantation is contraindicated. In each case, folliculogenesis and oogenesis are interrupted and long-term effects on the epigenome cannot be excluded, which could be due to extracorporeal treatment, exposure to potentially harmful substances, or the cryopreservation treatment itself. 

## 4. OTC and the Epigenome

The cryopreservation of mature oocytes or pre-implantation embryos, mostly via vitrification, is a very common and routinely applied MAR technique in humans. Survival and clinical outcomes are good; however, the adverse effects on epigenetic patterns are still of concern as several epigenome alterations have been reported in recent years, especially in animal models but also in humans [17,18,20,43,44,45]. To estimate the potential effect of the cryopreservation of immature (and not fully grown) oocytes, different phases must be considered: (i) immediate effects of cryopreservation directly after treatment, (ii) mid-term effects during in vivo/in vitro growth, and (iii) long-term effects in embryos/offspring. Moreover, since most cellular factors required for oocyte-to-embryo transition rely on maternal accumulation and storage in distinct compartments, such as the SCMC during oocyte growth, not just (i) DNA methylation patterns alone but also (ii) the abundance and availability of epigenome-related proteins and transcripts or (iii) post-translational histone modifications are of relevance [4,46,47]. 

### 4.1. Gene Expression and Protein Abundance 

In general, cryopreservation of ovarian tissue can result in changes in gene expression and protein abundance at different stages [48,49]. More precisely, cryopreservation also hampers several specific epigenetically relevant factors (Table 1). In mice, vitrification and warming of juvenile ovaries resulted in significantly reduced mRNA expression and protein abundance of DNMT1 [50]. Vitrification of fully grown immature ovine GV oocytes induced alterations in a subset of genes implicated in epigenetic control during oocyte maturation and early embryo development. These included DNA methyltransferase DNMT3B and histone deacetylase HDAC1 [51]. Slow freezing or vitrification of murine ovarian tissue followed by orthotopic transplantation also led to gene expression differences in the imprinted genes Igf2r, H19, and PLAGL1 in various tissue types in the offspring compared with natural controls [52]. In contrast, after vitrification of immature pre-antral follicles and subsequent in vitro follicle culture, the abundance of nearly 2000 proteins is similar between vitrified and non-vitrified controls in fully grown GV or MII oocytes [53]. This dataset also included several MEGs including MATER, histone acetyltransferase Hat1, and DNMT1. As a consequence, the cellular machinery establishing epigenetic patterns after vitrification of immature GV oocytes followed by in vitro growth might not be altered (or is even restored), although it is not representative of the entire proteome in mature oocytes [54]. Compared to the entire proteome, effects on lower-abundance proteins essential for epigenetic control cannot be excluded. 

### 4.2. DNA Methylation Patterns

The immediate effects of cryopreservation treatment without subsequent grafting or IVC were reported after vitrification and warming of juvenile murine ovaries. Here, the growth factor receptor-binding protein 10 (*Grb10*) promoter was hypermethylated compared with controls [50]. Similarly, 16 different single CpGs in the *Snrpn* DMR were analysed in GV oocytes from juvenile murine ovaries directly after cryopreservation and thawing. The *Snrpn* methylation status in vitrified/warmed GV oocytes did not vary from fresh controls [55]. Although allotransplantation was carried out afterwards, no epigenetic data regarding pre-implantation embryos or offspring were available. Certainly, the cryopreservation of fully grown GV oocytes followed by IVM is quite different to classic OTC approaches, especially as regards the unequal epigenetic status of early-stage GV and fully grown GV oocytes; however, freezing of GV oocytes might, in some cases, also be an option for fertility patients. Moreover, due to comparable chromatin organisation in early and fully grown GV oocytes (chromatin vs. condensed chromosomes), epigenetic effects after GV cryopreservation followed by IVM might be relevant at this point. Therefore, in brief, normal gDNA methylation patterns were observed in mouse MII oocytes after GV cryopreservation/IVM [56] and in the human imprinted genes *H19* and *KCNQ1OT1* [57]. In bovines, gDNA methylation was not affected after GV vitrification and IVM in MII oocytes, yet it was significantly reduced in pre-implantation embryos compared with fresh controls [58].

Some studies have been carried out in animal model systems concerning the mid-term effects during in vivo/in vitro growth or long-term effects in embryos/offspring. In a setup using vitrified and IVC murine pre-antral follicles, *H19*, *Igf2r*, and *Snrpn* imprinting patterns were analysed in fully grown GV oocytes. *H19* and *Igf2r* DNA methylation was comparable between in vivo/in vitro controls and the vitrified group, while some single CpG errors were reported in the maternally imprinted *Snrpn* gene [59]. Although indirect, different methylation patterns for *Inhba* and *Inhbb* were reported in mice after vitrification and IVC in granulosa cells from large antral follicles [60]. In the pioneering study of Sauvat et al. [61], two epigenetic marks in murine offspring were analysed for the first time after cryopreservation and grafting of immature tissue. No differences in the imprinted genes *H19* and *Lit1* were found in muscle, kidney, and tongue in offspring from grafted mice compared to controls. Similarly, a normal *Igf2r* methylation status was reported in ovine offspring after grafting of cryopreserved immature ovaries [62]. Controversially, in a recent study by Yan and colleagues [52], the methylation rates of four imprinted genes were analysed after slow freezing or vitrification of murine ovarian tissue followed by orthotopic transplantation. While the methylation pattern was stable in *Snrpn*, alterations were reported in *Igf2r*, *H19*, and *PLAGL1* in brain and liver tissue in the offspring compared with natural controls. These alterations were combined with different gene expression levels for Igf2r, H19, and PLAGL1, yet with no significant morphological/functional differences (e.g., birth defects, body weight gain, exercise capacity, or anti-fatigue ability) in offspring derived from either the cryopreserved or non-cryopreserved group. 

### 4.3. Post-Translational Histone Modifications

PTMs are also crucial for epigenetic control, and direct or indirect alterations of the histone landscape after cryopreservation could be of interest. Data regarding OTC and complete in vitro/in vivo resumption of gametogenesis are scarce. In one study, Tian et al. reported that H3K9me3, H3K4me3, and H3K27ac levels in murine pre-implantation embryos after cryopreservation and in vitro folliculogenesis were comparable to fresh controls [63]. When analysing PTMs after cryopreservation of immature GV, post-translational histone modifications (H3K9me3), either at the MII stage or in blastocysts, were not different after vitrification of immature bovine GV oocytes followed by in vitro maturation compared with fresh controls [58]. In contrast, in another study by Lee and Comizzoli [64], histone H3 trimethylation at lysine 4 (H3K4me3) was dramatically reduced after vitrification of immature GV oocytes in domestic cats compared with fresh controls, while H3K9me3 levels were unaffected. 

**Table 1:** Assessment of epigenome-related effects after OTC.

Reference	Type/Species (Cryopreservation Technique)	Analysis (Technique)	Main Outcome
Shirazi et al. (2016) [51]	Ovine GV oocytes, IVM (vitrification including 20% DMSO and 20% EG)	Epigenetically-relevant mRNA abundance in GV/embryos (real-time qPCR)	Alteration of DNMT3B and HDAC1
Demant et al. (2012) [53]	Murine pre-antral follicles, IVC (vitrification including 15% DMSO and 15% EG)	Proteome analysis in GV/MII (LC-MS/MS and 2D DIGE)	No differences between vitrified and non-vitrified GV/MII
He et al. (2018) [50]	Murine OTC (vitrification including 20% DMSO and 20% EG)	mRNA expression and protein abundance (Western blotting and real-time qPCR)	Decreased mRNA/protein levels for Dnmt1
Yan et al. (2020) [52]	Murine OTC and orthotopic transplantation (slow freezing including 1.5M DMSO; vitrification including 15% DMSO and 15% EG)	Epigenetically relevant mRNA abundance in offspring (real-time qPCR)	mRNA differences in H19, Igf2r and PLAGL1 but normal Snrpn expression
Yodrug et al. (2021) [58]	Bovine GV oocytes, IVM (vitrification including 15% DMSO and 15% EG)	Global DNA methylation in MII and embryos (immunofluorescent assay)	Normal gDNA pattern in MII but altered in blastocysts
Al-Khtib et al. (2011) [57]	Human GV oocytes, IVM (vitrification including PrOH and EG; concentrations n/a; DMSO free)	Imprinted genes in MII (bisulphite mutagenesis and sequencing)	Normal pattern for *H19* and *KCNQ1OT1*
Yan et al. (2014) [56]	Murine GV oocytes, IVM (vitrification including 15% PrOH and 15% EG; DMSO free)	Global DNA methylation in MII (immunofluorescent assay)	Normal gDNA pattern
Trapphoff et al. (2010) [59]	Murine pre-antral follicles, IVC (vitrification including 15% DMSO and 15% EG)	Imprinted genes in GV (bisulphite pyrosequencing)	Normal establishment of *H19* and *Igf2r* imprinting but some single CpG errors in *Snrpn*
Yan et al. (2020) [52]	Murine OTC and orthotopic transplantation (slow freezing including 1.5M DMSO; Vitrification including 15% DMSO and 15% EG)	Imprinted genes in offspring (MethylDetector PCR after bisulphite treatment)	Significant variations in *H19*, *Igf2r*, and *PLAGL1* but normal *Snrpn* methylation
He et al. (2018) [50]	Murine OTC (vitrification including 20% DMSO and 20% EG)	Methylation pattern (Western blotting and real-time qPCR; indirect)	Hypermethylation of the *Grb10* promoter region
Wang et al. (2013) [55]	Murine OTC (vitrification including 20% DMSO and 20% EG or 5.5M EG)	Methylation pattern in GV after vitrification/warming (bisulphite sequencing PCR)	Normal *Snrpn* methylation
Sauvat et al. (2008) [61]	Murine OTC and grafting (slow freezing including 1.5M DMSO)	Imprinted genes in offspring (Southern blotting)	Normal *H19* and *Lit1* methylation
Sauvat et al. (2013) [62]	OTC and grafting in ewes (slow freezing including 1.5M DMSO)	Imprinted gene in offspring (bisulphite mutagenesis and sequencing)	Normal *Igf2r* methylation
Damavandi et al. (2021) [60]	Murine pre-antral follicles, IVC (vitrification including 15% DMSO and 15% EG)	CpG methylation in granulosa cells (direct PCR sequencing after bisulphite treatment)	Altered *Inhba*/*Inhbb* methylation
Yodrug et al. (2021) [58]	Bovine GV oocytes, IVM (vitrification including 15% DMSO and 15% EG)	Histone modifications (immunofluorescent assay)	Normal H3K9me pattern in MII/blastocysts
Tian et al. (2022) [63]	Murine pre-antral follicles, IVC (vitrification including 0.75M EG and 0.75M PrOH)	Histone modifications in embryos (immunofluorescent assay)	Normal histone pattern (H3K9me3, H3K4me3, H3K27ac)
Lee and Comizzoli (2019) [64]	Domestic cat GV (vitrification including 20% DMSO and 20% EG)	Histone modifications after vitrification (immunofluorescent assay)	Normal H3K9me3 but altered H3K4me3

2D DIGE, two-dimensional difference gel electrophoresis; LC-MS/MS, liquid chromatography–mass spectrometry/mass spectrometry; qPCR, quantitative real-time PCR.

## 5. Testicular Tissue Cryopreservation (TTC)

**Figure 4:**
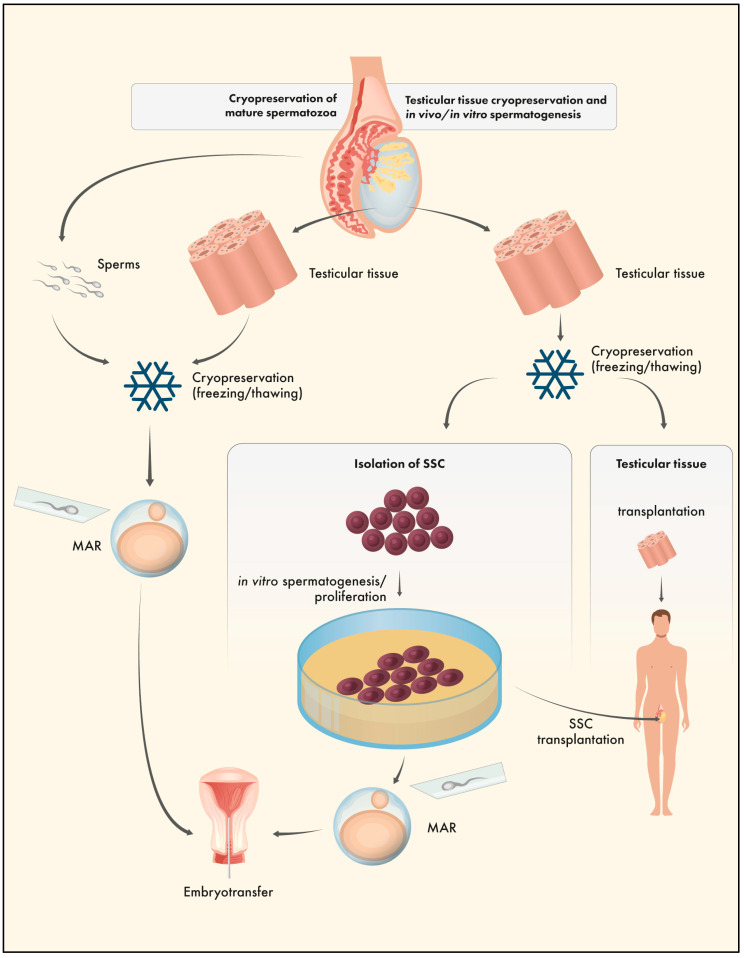
Strategies for fertility preservation in male patients. Cryopreservation of mature spermatozoa (left pathway) and (experimental) testicular tissue cryopreservation and in vivo/in vitro spermatogenesis (right pathway). Left pathway: Cryopreservation of mature spermatozoa or testicular tissue followed by medically assisted reproduction (MAR) techniques (including IVF, ICSI, TESE-ICSI). Right pathway: Testicular tissue cryopreservation followed by in vivo or in vitro spermatogenesis. Resumption of gametogenesis can occur after tissue grafting or, to avoid the risk of remission of malignant cells, after isolation of spermatogonial stem cells (SSC), in vitro proliferation/spermatogenesis, SSC transplantation, and/or MAR techniques.

In males, the option of choice for fertility preservation is the cryopreservation of mature spermatozoa. Sperm can be collected directly via ejaculation or more invasively after testicular biopsy. Later, mature sperms can be used for different MAR techniques including in vitro fertilisation, intracytoplasmic sperm injection, or, after testicular biopsy, via testicular sperm extraction followed by intracytoplasmic sperm injection (TESE-ICSI). Freezing/thawing of mature sperms has been used successfully in MAR (including IVF, ICSI, TESE-ICSI) for many decades [65]. However, mature spermatozoa can only be obtained after the onset of final spermatogenesis. In prepubertal cancer patients without active spermatogenesis, testicular tissue cryopreservation is the only option currently available prior to gonadotoxic radiotherapy or chemotherapy treatment. TTC offers the possibility of preserving spermatogonial stem cells (SSC) and resumption of gametogenesis, either in vivo or in vitro, for later treatment [66]. Males without active spermatogenesis have been offered testicular tissue banking for more than 20 years to potentially restore fertility after successful treatment (or provide the option to do so in the future). Comparable to OTC, the resumption of spermatogenesis can either occur in vivo after autotransplantation of thawed testicular tissue or isolated SSCs, or in vitro under appropriate culture conditions (Figure 4) [67,68,69]. Transplantation or culture of isolated SSC could avoid the remission of malignant cells and would be the better option for patients with metastatic malignancies or haematological cancer. Moreover, spermatogonial stem cell autotransplantation (SSCT) allows (so far only in animal model systems) the restoration of spermatogenesis in vivo (including natural conception without requiring MAR techniques); however, additional in vitro propagation is necessary to increase cell numbers. In contrast to tissue grafting, SSC isolation requires enzymatic digestion by collagenase and trypsin treatment or mechanical disaggregation; enzymatic treatment in particular might interfere with susceptibility to the cryopreservation process and cell viability [66,70]. 

Cryopreservation can be carried out via different protocols, including slow freezing or vitrification (or variants thereof); however, these protocols are still under development to optimise outcomes. Accordingly, in a mouse model, cryopreservation of testicular tissue might be more effective than testicular cell suspension cryopreservation [71]. Overall, the resumption of spermatogenesis in humans (and undoubtedly also in non-human model systems) is still challenging, and viable offspring have so far only been reported in animal model systems [72,73,74].

## 6. TTC and the Epigenome

Compared with OTC, TTC and resumption of gametogenesis, either in vivo or in vitro, is still in the developmental phase, and current research is mainly focused on the generation of healthy offspring (or the way towards it), mainly via fresh tissue or cells. Great progress has been made over the last two decades, yet studies including fresh/frozen and in vivo controls are still scarce and the assessment of the effects of cryopreservation might, at best, only constitute the next step. The need for proper controls is underlined by the fact that extracorporeal in vitro SSC propagation itself might induce epimutations [75]. Epigenetic instability was shown in several imprinted genes after cryopreservation of human testicular tissue and long-term IVC. Demethylation of paternally imprinted genes (*H19*, *H19*-DMR, and *MEG3*) along with increased methylation of maternally imprinted genes (*PEG3* and *KCNQ1OT*) were found during in vitro SSC culture. Controversially, a stable epigenetic profile was reported in mouse and marmoset SSC cultures without cryopreservation [76,77]. Whether epigenetic instability relies on cryopreservation or species differences remains unclear. Data regarding epigenetic effects after TTC must be considered with caution unless suitable controls are performed to distinguish between de novo epimutations due to cryopreservation or limited artificial spermatogenesis. 

In recent years, proof-of-principle regarding tissue/SSC transplantation was demonstrated for different techniques. Live offspring were reported after SSC autotransplantation and grafting or IVC of prepubertal testis in different species [72,73,78,79]. A normal DNA methylation pattern in the offspring was reported in mice after IVC of testicular tissue [79,80] or SSCT with fresh samples [73,81]. The more recent study by Serrano et al. found no major DNA methylation differences between SSCT-derived offspring and their corresponding controls. 

Since healthy mouse offspring with normal methylation patterns in several imprinted genes were produced after testicular tissue cryopreservation, thawing, culture, and fertilisation in vitro, one could speculate that cryopreservation of testicular tissue does not induce de novo epimutations per se [79,80]. This might be true for a single biological endpoint; however, epigenomic control is much more complex, being embedded in a dense epigenetic network, and (transient) alterations in intermediate stages cannot be excluded. So far, to the best of our knowledge, only two studies have assessed the direct influence of cryopreservation on the epigenetic profile (or factors related to it). Oblette et al. [82] cultured fresh and slow-frozen testicular tissue in vitro and compared them to in vivo controls (Table 2). Cryopreservation limited the spermatogenesis and fertilisation capacity, yet embryonic development was initiated after intracytoplasmic sperm injection. Post-translational histone modifications (H3K4me3, H3K27me3, H3K9ac) in embryos were comparable between spermatozoa generated in vitro and in vivo, while DNA differences in gDNA methylation were found after in vitro spermatogenesis (with or without cryopreservation) during early embryogenesis. In another study from the same group, different cryopreservation protocols (controlled slow freezing vs. solid surface vitrification) were also compared after IVC of fresh or frozen mouse prepubertal testes [83]. Relative mRNA levels of epigenetically relevant enzymes, including the DNA methyltransferases DNMT1 and DMT3A and several post-translational histone modifications (H3K4me3, H3K9ac, and H4K8ac), were altered compared with unfrozen or in vivo controls, while gDNA methylation was comparable in spermatozoa after cryopreservation. Unfortunately, the methylation pattern analysis of two imprinted genes (*H19* and *Igf2r*) was unsuccessful due to limited cell numbers. To date, there are no direct data regarding methylation patterns of imprinted genes following cryopreservation. However, cryopreservation might, therefore, pose a risk of inducing de novo epimutations; at which level, especially in later stages, remains unclear. 

It is noteworthy that common TTC cryopreservation protocols include DMSO as the main CPA [70]. As seen in different cell types, DMSO treatment can induce de novo epimutations, including misregulated expression of epigenetically relevant DNA methyltransferases, DNA hypo- or hypermethylation, or alterations in affected post-translational histone modifications [36,37,38,84]. Accordingly, cryopreservation of mature spermatozoa can lead to epimutations in *Igf2* in boar [85]; however, to date, no effects have been reported in humans in *Snrpn*, *Snurf*, *Ebe3a*, or *H19* [86,87]. Nevertheless, TTC might also require enzymatic digestion via trypsin/collagenase or mechanical disaggregation for SSC isolation, and this enzymatic treatment may also increase/potentiate susceptibility to the cryopreservation process of immature germ cells, including epigenetic alterations as seen after cryopreservation of mature spermatozoa in boar.

## 7. Conclusions—OTC and TTC and the Epigenome

Overall, data regarding epigenome patterns at different levels (imprinted genes, gDNA methylation, PTMs, or abundance of relevant transcripts and proteins) after OTC are controversial. While the epigenome, in most studies, presented (at best) no or only minor alterations, some studies revealed imprinting defects in pre-implantation embryos or offspring that could lead to detrimental imprinting disorders. This has to be taken into account, especially since several studies in humans have reported an increased risk of rare genomic imprinting disorders in children conceived by MAR techniques [19,23,24]. The cryopreservation of mature MII oocytes or pre-implantation embryos can lead to epimutation, as seen in several studies; this effect can certainly also be transferred to immature oocytes, as highlighted herein. OTC affords the possibility of restoring initial (or transient) epimutations after subsequent growth and differentiation; however, long-term effects cannot be excluded. Additionally, it has to be considered that epigenetic control is normally not an all-or-nothing mechanism. Single CpG errors or (slightly) reduced DNA methylation patterns may not ultimately lead to imprinting disorders as there is a great distance between (epigenetic) genotype and (functional) phenotype. As for OTC, differences between studies might be due to different cell stages (prepubertal vs. adult) or cell composition (isolated follicles vs. tissue), different species, or methods to resume gametogenesis (in vitro vs. in vivo), different cryopreservation protocols (vitrification vs. slow freezing) and CPA compositions (DMSO free vs. DMSO/EG), or the sensitivity of the analysis tools (bisulphite treatment and pyrosequencing vs. Southern blotting). Although OTC and autotransplantation has been applied increasingly to preserve fertility in female patients, studies addressing the question of OTC and imprinting disorders after cryopreservation are rare, particularly in humans, and further research is needed to exclude any potential long-lasting adverse effects derived from cryopreservation techniques. 

In contrast to OTC, testicular tissue cryopreservation and resumption of spermatogenesis, either in vivo or in vitro, must still be considered an experimental approach in humans. However, testicular tissue transplantation affords the possibility of restoring fertility and producing healthy offspring in model systems. No major epigenetic alterations in offspring were reported, although minor changes were present. Artificial spermatogenesis per se could pose a risk of epimutations, even without cryopreservation, thus, making the estimation of environmental effects caused by cryopreservation on the epigenome quite difficult. Few studies in the literature have addressed whether the effects of enzymatic treatment, CPA exposure, processing for cryopreservation treatment, or cryopreservation itself interfere with the epigenome. Since TTC is still an experimental approach, it is evident that further research is needed to assess the potential, long-lasting, adverse effects of testicular tissue cryopreservation per se before the transition to human clinical trials can occur. This especially includes potential epimutations. 

## Figures and Tables

**Table 2:** Assessment of epigenome-related effects after TTC.

Reference	Type/Species (Cryopreservation Technique)	Analysis (Technique)	Main Outcome
Oblette et al. (2019) [83]	Murine in vitro culture of TT (solid surface vitrification including 2.1 mol/L DMSO and 2.7 mol/L EG or slow freezing including 1.5 mol/L DMSO)	Testicular tissue after IVC (qPCR and immunofluorescent assay)	Normal expression of epigenetic modification enzymes and gDNA methylation, but differences in histone modification
Oblette et al. (2021) [82]	Murine in vitro culture of TT (slow freezing including 1.5M DMSO)	Pre-implantation embryo (immunofluorescent assay)	Normal post-translational histone modifications and altered gDNA methylation

## Data Availability

Not applicable.
